# Unemployment persistence in Europe: evidence from the 27 EU countries

**DOI:** 10.1016/j.heliyon.2022.e08898

**Published:** 2022-02-02

**Authors:** Guglielmo Maria Caporale, Luis A. Gil-Alana, Pablo Vicente Trejo

**Affiliations:** a Brunel University London, UK; b University of Navarra, Spain; c University of Huelva, Huelva, Spain

**Keywords:** Unemployment persistence, Long memory, Europe, Fractional integration

## Abstract

This paper investigates unemployment persistence in the 27 EU member states by applying fractional integration methods to quarterly data (both seasonally adjusted and unadjusted) from 2000q1 to 2020q4. The obtained evidence points to high levels of persistence in all cases. With seasonally adjusted data, a small degree of mean reversion is found in the case of Belgium, Luxembourg and Malta, but this evidence disappears under the assumption of weakly correlated disturbances. More cases of mean reversion are found instead when analysing the unadjusted series. In particular, countries such as Belgium, France, Croatia, Italy, Luxembourg and Malta display orders of integration significantly lower than 1. In addition, significant negative time trends are found in the case of Bulgaria, Croatia, Malta and Romania, and a positive one for Luxembourg. Finally, the Covid-19 pandemic had mixed effects, with (seasonal) persistence increasing in some countries whilst decreasing in others and not changing in a minority of cases. On the whole, our results support the hysteresis hypothesis for the European economies.

## 1 Introduction

Unemployment persistence is an issue that has attracted considerable attention over the years given its implications for the real economy, general welfare, policy design, and also the empirical relevance of the two main existing unemployment theories. In particular, according to the natural rate hypothesis (Phelps, 1968; Friedman, 1968), unemployment should be a stationary and mean-reverting process, i.e. the differencing parameter d should be 0. Therefore, exogenous shocks should only have transitory effects; more specifically, they should only lead to temporary deviations from the long-run equilibrium level known as the natural rate, where the speed of adjustment is an issue to be analyzed empirically. This view belongs to a neoclassical (monetarist) interpretation of the functioning of the economic system, and it is used to argue against the intervention of governments and/or Central Banks to keep the unemployment rate under control. Instead in New Keynesians the unemployment rate may converge towards a so-called NAIRU (non-accelerating rate of unemployment) in the long run; however, in the short run some deviations may exist and persist due to nominal rigidity, and thus there is an argument for demand management policies to mitigate them.

An alternative framework is the hysteresis model developed by Blanchard and Summers (1986, 1987) in which mean reversion does not necessarily occur – factors such as the presence of strong unions or the stigma attached to long-term unemployment can result in the d parameter being equal to or above 1, which implies that exogenous shocks will have permanent effects. Various theoretical models have been put forward to shed light on the possible determinants of hysteresis and/or endogenise the natural rate of unemployment. The factors considered include in turn productivity growth (Pissarides, 1990), real interest rates (Blanchard, 1999), stock prices (Phelps, 1999), institutional variables (Nickell, 1998; Nickell and Van Ours, 2000), or the interaction between institutional and macroeconomic variables (Blanchard and Wolfers, 2000). Most of the available empirical evidence suggests that the NAIRU model is more appropriate for the US whilst the hysteresis one is a better match for the Europe, the latter being characterized by more labour market rigidities – in fact over the period 2000–2020 the average unemployment rate in Europe was 9.1%, was higher than 10% in countries such as Bulgaria, Spain, Greece, Croatia, Lithuania, Latvia, Poland and Slovakia, and above 15% in Spain and Greece.

The aim of the current study is to assess the degree of unemployment persistence in the 27 European Union (EU) member states (and whether the Covid-19 pandemic had affected it) by using fractional integration methods to estimate the (possibly fractional) differencing parameter d. This parameter represents the order of integration of the series of interest, and in the framework used here it is allowed to take not only integer values (as in the standard approach based on the dichotomy between I(0) and I(1) series), but also any fractional value between 0 and 1 or even above 1. This more general modelling approach incorporates a variety of stochastic processes and provides information about whether or not mean reversion occurs as well as the speed of adjustment towards the long-run equilibrium; the latter also represents the degree of persistence of the series and sheds light on the permanent or transitory nature of the effects of exogenous shocks. Note that the terms hysteresis and persistence are used interchangeably in most studies in the unemployment literature. However, an alternative view exists according to which unit root (or near unit root) dynamics are only an approximation to the memory characteristics of hysteresis which is theoretically different and also not observationally equivalent. More specifically, hysteresis is thought to be a property of an input-output system whilst persistence concerns univariate processes; in particular, unit root processes can exhibit persistence but not remanence (see Amable et al., 1993, 1994) and have a long or unselective memory of every past shock (see Göcke 2002). By contrast, in a system characterised by hysteresis the output does not depend on all past values of the input but only on the non-dominated maxima and minima. We are aware of this view but in the current paper follow the common practice of considering hysteresis and persistence equivalent terms.

The paper is structured as follows: Section 2 reviews the literature on unemployment persistence in Europe; Section 3 outlines the methodology; Section 4 describes the data and the empirical results; Section 5 provides some concluding remarks.

## 2 Literature review

The empirical literature on modelling the unemployment rate has expanded considerably since the eighties when Blanchard and Summers (1986) published an influential paper estimating an unemployment equation with a lagged term, a time trend and a moving average component in the error term, and provided evidence of much higher hysteresis in Europe relative to the US. In a follow-up study Alogoskoufis and Manning (1988) investigated unemployment persistence in the OECD countries by using an extended version of the Blanchard and Summers (1986) model of wage and employment setting; again, they found high unemployment persistence in Europe and attributed the increase in the natural rate of unemployment to the sluggishness in European labour demand.

Barro (1988) measured unemployment persistence using the estimated coefficients of an AR(1) model, and argued that unionization and government size increase persistence in the countries where corporatism is not present. Other studies have estimated more general AR(p) models (e.g., Son et al., 2010), panel quantil regressions (Andini and Andini, 2015), and dynamic panel data models (Arulampalam et al., 2000), and obtained similar evidence of high unemployment persistence.

A more recent strand of the literature uses fractional integration methods. For instance, Gil-Alana and Henry (2003) estimated a fractionally integrated ARMA model for UK unemployment and found that the unit root hypothesis is decisively rejected, and that including regressors such as real oil price and real interest rate produces estimates of the parameter d between 0.5 and 1, which implies that the UK unemployment is mean-reverting, but the effects of shocks are long-lived. Caporale and Gil-Alana (2007) concluded that a hysteresis model with path dependency is more suitable than a NAIRU framework for US unemployment; according to their results, in the case of the US there is no constant long-run equilibrium rate, the effects of exogenous shocks do not die away within a finite time horizon, and unemployment is nonstationary. Caporale and Gil-Alana (2008) investigated the issues of fractional integration and structural breaks for US, UK and Japanese unemployment; they found that a structuralist interpretation is more appropriate for the US and Japan, whilst a hysteresis model works better for the UK.

Figuereido (2010) studied Brazilian unemployment dynamics using fractional integration. His results suggest that unemployment is a non-stationary variable; however, mean reversion occurs at the regional level. Cuestas et al. (2011) analyzed unemployment hysteresis, structural changes, non-linearities and fractional integration in various European transition economies. Their unit root test results imply non-stationarity of the unemployment series in most of the countries under examination, which would support the hysteresis hypothesis. However, the evidence based on fractional integration methods suggests instead that mean reversion occurs, consistently with the NAIRU hypothesis, in a number of cases.

Shalari et al. (2015) applied fractional integration methods to analyze Albanian unemployment and found asymmetries (specifically, negative shocks have a bigger impact than positive ones) but no persistence. Caporale and Gil-Alana (2018) provided evidence of asymmetric behaviour in Spanish unemployment; specifically, the degree of persistence is higher during recessions than during expansions; in both cases the estimates of d are higher than 1, which represents evidence of hysteresis. More papers using fractional integration and long-memory models to analyse unemployment rates include those by Tscherning and Zimmermann (1992), Koustas and Veloce (1996), Van Dijk and Franses (2002), Komornik and Komornikova (2005), Lahiani and Scaillet (2009), Kurita (2010), Shalari et al. (2015), Leites and Porras (2016), Tule et al. (2017), etc.

## 3 Methodology

As mentioned before, various studies have measured persistence using the estimated coefficient of a simple AR(1) process, or the sum of the coefficients in a general AR(p) model. However, such methods are based on a dichotomy between I(0) and I(1) series which produces an abrupt change in the behaviour of the series depending on whether or not a unit root is incorporated in the model, and it is well known that standard unit root tests have extremely low power if the processes are in fact fractionally integrated (Diebold and Rudebusch, 1991; Hassler and Wolters, 1994; Lee and Schmidt, 1996; Ben Nasr et al., 2014; etc.). For this reason, we use instead a fractional integration (long memory) approach.

A process is said to be fractionally integrated or integrated of order d (denoted as I(d)) if it can be expressed as:

(1) $$\;{\left(1\;-\;B\right)}^{d}x\left(t\right)\;=\;u\left(t\right),\;t\;=\;1\;,\;2\;,\;...\;,$$

where B is the backshift operator, i.e., B^p^x(t) = x(t-p), and u(t) is integrated of order 0 or I(0) (also named short memory) and is a covariance stationary process which is characterized by a finite sum of its autocovariances and includes, for instance, the stationary AR(MA) processes. Note that if u(t) is an ARMA(p, q) process, x(t) in (1) becomes an AutoRegressive Fractionally Integrated Moving Average, ARFIMA(p, d, q) process.

We estimate the differencing parameter d by using a testing approach due to Robinson (1994) which is based on the Whittle function in the frequency domain. This method is quite flexible because it allows the inclusion of deterministic terms such as an intercept and a time trend and is not constrained to the stationary range for the values of d (d < 0.5); moreover, it has a standard normal N(0,1) limit distribution which holds independently of the way of modelling the I(0) error term, and it is the most efficient method in the Pitman sense against local departures from the null. A full description can be found in Gil-Alana and Robinson (1997).

## 4 Data and empirical results

We analyze the unemployment rate in the 27 EU member states, namely Austria, Belgium, Bulgaria, Cyprus, Czech Republic, Denmark, Estonia, Spain, Finland, France, Greece, Croatia, Hungary, Ireland, Italy, Lithuania, Luxembourg, Latvia, Malta, Netherlands, Poland, Portugal, Romania, Sweden, Slovenia and Slovakia; in addition, we examine the EU mean rate. The series are quarterly and cover the period from 2000q1to 2020q4. We use both the seasonally adjusted and the raw, unadjusted data. The source is the Eurostat database, in particular the appendix called “Unemployment by sex and age (1992–2020) – quarterly data”.

Table 1 displays some descriptive statistics for the seasonally adjusted series. It is immediately apparent that there are significant differences between the unemployment rate in the various EU member states. The maximum value ranges between 6.2% in Austria and 27.6% in Greece; the mean for the EU is 11.4%. The range for the minimum values is much narrower, namely between 2% in the Czech Republic and 8% in Spain; the mean for the EU is 6.5%. As for the mean unemployment rate, it ranges between 4.7% in the Netherlands and 15.9% for Spain; the corresponding value for the EU is 9.1%. Volatility, as measured by the standard deviation, also varies considerably across countries, ranging between 0.46% in Belgium and 6.60% in Greece, with a value of 1.27% for the EU series.

**Table 1:** Descriptive statistics. Seasonally Adjusted Data.

Country	Maximum	Minimum	Mean	Std. Dev.
AT	6.2	3.4	4.92	0.60
BE	8.8	5.1	7.49	0.46
BG	20.7	4.1	10.25	4.21
CY	16.6	3.3	7.84	4.04
CZ	9.2	2	5.94	2.87
DE	11.3	3.1	6.78	2.22
DK	8.2	3.4	5.66	0.89
EE	19.3	4	8.91	3.20
ES	26.3	8	15.9	4.95
EU	11.4	6.5	9.1	1.27
FI	10.5	6.2	8.23	0.55
FR	10.1	6.8	8.72	1.10
GR	27.6	7.6	15.71	6.60
HR	18.1	6.3	12.64	2.74
HU	11.4	3.4	7.07	2.77
IE	16	4	8.07	4.14
IT	12.8	6	9.43	1.33
LT	18.1	4.1	10.62	4.04
LU	7.8	1.9	4.89	1.15
LV	20.9	5.4	11.23	4.23
MT	8.3	3.5	5.96	1.06
NL	7.8	2.2	4.7	0.99
PL	20.4	2.9	10.82	4.28
PT	17.3	3.8	9.08	3.31
RO	9.2	3.8	6.51	1.26
SE	8.9	4.7	6.95	1.24
SI	10.6	4.1	6.78	1.17
SK	19.3	5.7	12.97	4.05

AT: Austria, BE: Belgium, BG: Bulgaria, CY: Cyprus, CZ: Czech Republic, DE: Germany, DK: Denmark, EE: Estonia, ES: Spain, EU: European Union, FI: Finland, FR: France, GR: Greece, HR: Croatia, HU: Hungary, IE: Ireland, IT: Italy, LT; Lithuania, LU: Luxembourg, LV: Latvia, MT: Malta, NL: Netherlands, PL: Poland, PT: Portugal, RO: Romania, SE: Sweden, SI: Slovenia, SK: Slovakia.

Table 2 reports the same information for the unadjusted series. There is again a wide range of maximum values (the difference between the highest and the lowest is 21.6%), and a narrower one for the minimum values (the corresponding difference is 6.1%). The lowest mean value is found in the Netherlands (4.7%) and the highest in Spain (15.9%); the EU mean value is 9.11%. There is also a wide volatility range, the lowest standard deviation being 0.50% in France and the highest 6.28% in Poland. The Southern countries are clearly characterized by higher (though not more volatile) unemployment.

**Table 2:** Descriptive statistics. Seasonally Unadjusted Data.

Country	Maximum	Minimum	Mean	Std. Dev.
AT	6.3	3.1	4.92	0.87
BE	9.1	4.9	7.49	0.58
BG	22.3	3.7	10.27	4.86
CY	17.7	3.2	7.84	5.07
CZ	9.6	1.9	5.94	2.38
DE	11.5	3.1	6.78	1.34
DK	8.5	3.3	5.67	1.51
EE	19.5	3.9	8.91	2.76
ES	26.9	7.9	15.9	5.48
EU	12	6.4	9.11	1.75
FI	11.1	5.6	822	1.66
FR	10.5	6.7	8.72	0.50
GR	27.9	7.3	15.71	5.97
HR	18.7	5.7	12.65	3.33
HU	11.9	3.3	7.07	1.75
IE	15.9	3.8	8.08	4.26
IT	13.6	5.6	9.43	1.78
LT	18.2	3.8	10.62	4.52
LU	7.9	1.8	4.88	0.55
LV	21.3	5.3	11.23	3.98
MT	8.3	3.5	5.96	1.34
NL	8.1	2.1	4.7	1.14
PL	20.7	2.9	10.83	6.28
PT	17.8	3.8	9.08	2.93
RO	10.3	3.8	6.52	0.38
SE	9.5	4.7	6.96	1.31
SI	11.1	4	6.78	0.95
SK	19.9	5.6	12.98	3.46

AT: Austria, BE: Belgium, BG: Bulgaria, CY: Cyprus, CZ: Czech Republic, DE: Germany, DK: Denmark, EE: Estonia, ES: Spain, EU: European Union, FI: Finland, FR: France, GR: Greece, HR: Croatia, HU: Hungary, IE: Ireland, IT: Italy, LT; Lithuania, LU: Luxembourg, LV: Latvia, MT: Malta, NL: Netherlands, PL: Poland, PT: Portugal, RO: Romania, SE: Sweden, SI: Slovenia, SK: Slovakia.

We estimate the following regression model:

(2) y(t)=α+βt+x(t),t=1,2,...

where x(t) is assumed to be integrated of order d, or I(d) i.e.,

(3) $${\left(1\;-\;B\right)}^{d}x\left(t\right)\;=\;u\left(t\right)\;,\;t\;=\;1\;,\;2\;,\;...\;,$$

where u(t) is I(0) or a short-memory process. Then, if d > 0, x(t) displays long memory, i.e. observations are highly dependent even if they are far apart in time, with higher values of d indicating stronger dependence.

Table 3 – 5 display the estimates of d along with their 95% confidence bands corresponding to three model specifications: i) no deterministic terms, ii) a constant, and iii) a constant and a linear time trend. The values in bold are those from the model selected in each case on the basis of the statistical significance of the regressors. Note that Eqs. (2) and (3) can be jointly represented as

(4) $$\tilde{y}\left(t\right)\;=\;\alpha \;\tilde{1}\left(t\right)\;+\;\beta \;\tilde{t}\left(t\right)\;+\;u\left(t\right)\;,\;t\;=\;1\;,\;2\;,\;...\;$$

where

$$\tilde{y}\left(t\right)\;=\;{\left(1\;-\;B\right)}^{d}y\left(t\right);\;\tilde{1}\left(t\right)\;=\;{\left(1\;-\;B\right)}^{d}1;\;\tilde{t}\left(t\right)\;=\;{\left(1\;-\;B\right)}^{d}t$$

and since u(t) is I) (0) by assumption, standard t-values are valid.

**Table 3:** Seasonally adjusted data. Results based on white noise errors.

Countries	No terms	A constant	A constant and a linear trend
AT	0.90 (0.77, 1.06)	**0.91 (0.77, 1.13)**	0.91 (0.76, 1.13)
BE	0.89 (0.78, 1.05)	**0.81 (0.71, 0.95)∗**	0.81 (0.70, 0.95)
BG	1.04 (0.92, 1.21)	**1.23 (1.11, 1.38)**	1.23 (1.11, 1.38)
CY	1.28 (1.14, 1.46)	**1.54 (1.39, 1.75)**	1.54 (1.39, 1.75)
CZ	1.03 (0.89, 1.23)	**1.67 (1.48, 1.90)**	1.67 (1.48, 1.89)
DE	1.04 (0.93, 1.20)	**1.41 (1.31, 1.54)**	1.41 (1.31, 1.54)
DK	1.01 (0.88, 1.18)	**1.20 (1.07, 1.38)**	1.20 (1.07, 1.38)
EE	0.98 (0.84, 1.15)	**1.11 (0.96, 1.32)**	1.11 (0.96, 1.31)
ES	1.07 (0.94, 1.25)	**1.73 (1.59, 1.94)**	1.73 (1.58, 1.93)
EU	0.98 (0.85, 1.16)	**1.59 (1.43, 1.82)**	1.58 (1.43, 1.79)
FI	0.93 (0.80, 1.11)	**1.20 (1.04, 1.43)**	1.20 (1.04, 1.42)
FR	0.87 (0.73, 1.06)	**1.14 (1.01, 1.34)**	1.14 (1.01, 1.33)
GR	1.10 (0.98, 1.27)	**1.61 (1.51, 1.74)**	1.61 (1.51, 1.73)
HR	1.08 (0.94, 1.26)	**1.21 (1.10, 1.37)**	1.21 (1.10, 1.37)
HU	1.05 (0.93, 1.21)	**1.24 (1.14, 1.37)**	1.24 (1.14, 1.37)
IE	1.24 (1.13, 1.38)	**1.47 (1.36, 1.62)**	1.47 (1.36, 1.62)
IT	0.90 (0.76, 1.10)	**1.21 (1.11, 1.34)**	1.21 (1.11, 1.33)
LT	1.12 (0.99, 1.30)	**1.55 (1.40, 1.76)**	1.55 (1.40, 1.76)
LU	0.32 (0.22, 0.61)	0.48 (0.41, 0.58)	**0.41 (0.30, 0.55) ∗ (+)**
LV	1.09 (0.95, 1.28)	**1.34 (1.20, 1.53)**	1.34 (1.20, 1.52)
MT	0.97 (0.85, 1.13)	0.80 (0.70, 0.95)	**0.77 (0.63, 0.95)∗ (-)**
NL	1.14 (1.00, 1.30)	**1.42 (1.27, 1.64)**	1.42 (1.27, 1.64)
PL	1.08 (0.95, 1.24)	**1.52 (1.39, 1.69)**	1.51 (1.39, 1.69)
PT	1.13 (1.04, 1.26)	**1.30 (1.20, 1.43)**	1.29 (1.20, 1.43)
RO	0.97 (0.84, 1.16)	**0.88 (0.73, 1.12)**	0.88 (0.69, 1.12)
SE	0.94 (0.82, 1.11)	**1.21 (1.04 1.43)**	1.21 (1.04, 1.43)
SI	1.01 (0.87, 1.19)	**1.12 (1.02, 1.25)**	1.12 (1.02, 1.25)
SK	1.06 (0.93, 1.25)	**1.78 (1.60, 2.03)**	1.80 (1.60, 2.05)

The reported values are the estimates of d. In parenthesis, the 95% confidence intervals. In bold, the selected specification according to the deterministic terms. ∗: evidence of mean reversion at the 95% level; (+) indicates a positive time trend and (-) a negative time trend.

Table 3 reports the results for the seasonally adjusted series under the assumption of white noise errors. The time trend coefficient is found to be significant only for a couple of countries, namely Luxembourg, with a positive trend, and Malta, with a negative one. The estimated values of d are relatively large in all cases, and imply the presence of long memory in all the series examined. Evidence of mean reversion, i.e., d < 1 is found for Luxembourg (d = 0.41), Malta (0.77) and Belgium (0.81); in all the other cases, the estimated values of d are equal to or higher than 1. The unit root null hypothesis (d = 1) cannot be rejected for Romania (0.88), Austria (0.91) and Estonia (1.11); in all the other cases d is significantly higher than 1.

Table 4 displays the corresponding results under the assumption of (Bloomfield) autocorrelated errors. Bloomfield (1973) showed that the log-spectral density function of ARMA processes could be fairly well approximated using a simple expression with very few parameters. It is a non-parametric way of modelling autocorrelated I(0) errors since no functional form is present. Using this approach, again, only Luxembourg and Malta exhibit significant trends (positive and negative respectively), but evidence of mean reversion is now not found in any single case. In fact, although some estimates of d are below 1, the confidence intervals are now wider and include the unit root (d = 1) in all cases. The implication is that shocks have long-lived effects – in other words the series are characterized by very high levels of persistence.

**Table 4:** Seasonally adjusted data. Results based on autocorrelated (Bloomfield) errors.

Countries	No terms	A constant	A constant and a linear trend
AT	1.06 (0.73, 1.47)	**0.75 (0.37, 1.27)**	0.77 (0.44, 1.27)
BE	1.04 (0.80, 1.46)	**1.07 (0.82, 1.42)**	1.07 (0.80, 1.42)
BG	1.12 (0.83, 1.53)	**1.49 (1.17, 1.87)**	1.49 (1.17, 1.89)
CY	1.28 (0.99, 1.70)	**1.37 (1.12, 1.73)**	1.37 (1.12, 1.74)
CZ	0.97 (0.71, 1.35)	**1.34 (0.88, 2.00)**	1.38 (0.89, 2.04)
DE	1.11 (0.90, 1.38)	**1.92 (1.57, 2.23)**	1.92 (1.57, 2.29)
DK	1.12 (0.79, 1.53)	**1.21 (0.86, 1.62)**	1.21 (0.87, 1.68)
EE	1.13 (0.76, 1.63)	**0.97 (0.66, 1.42)**	0.97 (0.63, 1.42)
ES	1.06 (0.79, 1.49)	**1.66 (1.37, 2.16)**	1.67 (1.37, 2.10)
EU	0.98 (0.72, 1.36)	**1.53 (1.23, 2.06)**	1.55 (1.24, 2.02)
FI	0.98 (0.70, 1.43)	**1.14(0.79, 1.70)**	1.14 (0.79, 1.74)
FR	0.85 (0.53, 1.27)	**1.25 (1.00, 1.68)**	1.25 (1.01, 1.72)
GR	1.20 (0.92, 1.55)	**2.02 (1.75, 2.45)**	2.03 (1.76, 2.46)
HR	1.07 (0.76, 1.48)	**1.33 (1.06, 1.67)**	1.33 (1.06, 1.67)
HU	1.07 (0.86, 1.36)	**1.44 (1.21, 1.79)**	1.45 (1.22, 1.80)
IE	1.50 (1.24, 1.87)	**1.71 (1.47, 2.14)**	1.74 (1.47, 2.14)
IT	0.87 (0.53, 1.30)	**1.55 (1.33, 1.95)**	1.54 (1.32, 1.88)
LT	1.17 (0.84, 1.58)	**1.44 (1.09, 1.87)**	1.44 (1.09, 1.88)
LU	0.98 (0.50, 1.33)	0.59 (0.41, 1.11)	**0.71 (0.41, 1.11) (+)**
LV	1.06 (0.73, 1.53)	**1.40 (1.01, 2.10)**	1.40 (1.01, 2.11)
MT	1.10 (0.83, 1.46)	0.80 (0.64, 1.07)	**0.72 (0.40, 1.07) (-)**
NL	1.35 (1.00, 1.87)	**1.21 (0.93, 1.56)**	1.21 (0.95, 1.56)
PL	1.08 (0.86, 1.41)	**1.46 (1.18, 1.80)**	1.46 (1.19, 1.76)
PT	1.63 (1.35, 2.33)	**1.58 (1.36, 1.89)**	1.60 (1.36, 1.91)
RO	0.91 (0.64, 1.29)	0.66 (0.47, 1.03)	**0.58 (0.32, 1.03) (-)**
SE	1.08 (0.79, 1.52)	**1.12 (0.60, 1.74)**	1.12 (0.70, 1.80)
SI	0.97 (0.69, 1.35)	**1.40 (1.14, 1.74)**	1.40 (1.14, 1.77)
SK	1.00 (0.75, 1.37)	**1.45 (1.08, 2.00)**	1.45 (1.09, 2.02)

The reported values are the estimates of d. In parenthesis, the 95% confidence intervals. In bold, the selected specification according to the deterministic terms. ∗: evidence of mean reversion at the 95% level; (+) indicates a positive time trend and (-) a negative time trend.

The above results are based on seasonally adjusted data. However, it is well known that seasonal adjustment can cause a significant loss of valuable information about the behavior of time series and also result in invalid inference about their relationships (see Ghysels, 1988; Barksy and Miron, 1989; Chatterjee and Ravikumar, 1992; Braun and Evans, 1995, among others). Therefore, next we re-estimate the models using the unadjusted series. Given the quarterly frequency of the data we assume that u(t) in (3) follows a seasonal (quarterly) AR(1) which can be specified as:

(5) u(t)=φu(t−4)+ε(t),t=1,2,...

where ε(t) is now a white noise process. Table 5 reports the estimates for the three different specifications, those in bold corresponding to the selected models; Table 6 provides more details for the latter. It can be seen that now the time trend is significant in a higher number of cases; again the only positive one is found in the case of Luxembourg, but negative ones are now estimated in the case of Bulgaria, Croatia, Malta and Romania. As for the estimates of d, these imply that mean reversion occurs in Belgium, Croatia, France, Italy, Luxembourg and Malta. The unit root null cannot be rejected for Austria, Bulgaria, Germany, Denmark, Estonia, Finland, Hungary, Ireland, Netherlands, Portugal, Romania, Sweden and Slovenia, while for the rest of the countries (Czech Republic, Spain, European Union, Greece, Lithuania, Latvia, Poland and Slovakia) the estimated value of d is found to be significantly higher than 1.

**Table 5:** Seasonally unadjusted data. Results based on seasonal AR(1) errors.

Countries	No terms	A constant	A constant and a linear trend
AT	0.64 (0.46, 0.84)	**0.70 (0.50, 1.02)**	0.68(0.48, 1.02)
BE	0.81 (0.63, 0.98)	**0.66 (0.54, 0.81)∗**	0.66 (0.53, 0.81)
BG	0.84 (0.70, 1.05)	0.90 (0.75, 1.12)	**0.90 (0.75, 1.12) (-)**
CY	1.00 (0.83, 1.24)	**1.06 (0.89, 1.32)**	1.06 (0.89, 1.31)
CZ	0.93 (0.74, 1.13)	**1.37 (1.11, 1.70)**	1.37 (1.11, 1.68)
DE	0.89 (0.67, 1.09)	**1.02 (0.87, 1.23)**	1.02 (0.87, 1.22)
DK	0.81 (0.65, 1.02)	**0.93 (0.75, 1.18)**	0.93 (0.75, 1.18)
EE	0.95 (0.80, 1.13)	**1.07 (0.91, 1.28)**	1.07 (0.91, 1.28)
ES	1.05 (0.89, 1.23)	**1.39 (1.21, 1.65)**	1.38 (1.20, 1.62)
EU	0.85 (0.64, 1.08)	**1.34 (1.07, 1.73)**	1.32 (1.07, 1.63)
FI	0.76 (0.56, 1.00)	**1.10 (0.80, 1.45)**	1.10 (0.81, 1.45)
FR	0.76 (0.52, 0.98)	**0.66 (0.46, 0.96)∗**	0.68 (0.46, 0.96)
GR	0.91 (0.75, 1.13)	**1.19 (1.06, 1.36)**	1.19 (1.06, 1.36)
HR	0.97 (0.76, 1.24)	0.80 (0.69, 0.97)	**0.79 (0.69, 0.97)∗ (-)**
HU	0.93 (0.78, 1.15)	**0.96 (0.81, 1.20)**	0.96 (0.81, 1.20)
IE	1.03 (0.89, 1.20)	**1.14 (1.00, 1.33)**	1.14 (1.00, 1.33)
IT	0.81 (0.59, 1.06)	**0.69 (0.55, 0.88)∗**	0.69 (0.55, 0.88)
LT	0.99 (0.82, 1.19)	**1.28 (1.09, 1.56)**	1.28 (1.09, 1.55)
LU	0.32 (0.21, 0.59)	0.46 (0.37, 0.57)	**0.38 (0.25, 0.54)∗ (+)**
LV	1.06 (0.90, 1.25)	**1.29 (1.13, 1.50)**	1.29 (1.13, 1.50)
MT	0.96 (0.78, 1.13)	0.79 (0.67, 0.96)	**0.76 (0.61, 0.95)∗ (-)**
NL	0.92 (0.76, 1.11)	**1.22 (1.00, 1.54)**	1.22 (1.00, 1.52)
PL	0.88 (0.69, 1.12)	**1.27 (1.07, 1.56)**	1.27 (1.07, 1.55)
PT	0.98 (0.84, 1.15)	**1.06 (0.93, 1.24)**	1.06 (0.93, 1.24)
RO	0.73 (0.54, 0.95)	0.63 (0.44, 1.04)	**0.65 (0.40, 1.04) (-)**
SE	0.67 (0.48, 0.88)	**1.05 (0.76, 1.46)**	1.05 (0.74, 1.43)
SI	0.88 (0.70, 1.11)	**0.85 (0.71, 1.04)**	0.85 (0.71, 1.04)
SK	0.95 (0.74, 1.18)	**1.31 (1.07, 1.70)**	1.32 (1.07, 1.70)

The reported values are the estimates of d. In parenthesis, the 95% confidence intervals. In bold, the selected specification according to the deterministic terms. ∗: evidence of mean reversion at the 95% level; (+) indicates a positive time trend and (-) a negative time trend.

**Table 6:** Estimated coefficients from the selected models in Table 5.

Country	d	Seasonal	Intercept	Time trend
AT	0.70 (0.50, 1.02)	0.418	4.3701 (10.58)	–
BE	0.66 (0.54, 0.81)	0.452	7.1840 (17.58)	–
BG	0.90 (0.75, 1.12)	0.471	18.7062 (18.62)	-0.1603 (-2.16)
CY	1.06 (0.89, 1.32)	0.729	5.5402 (7.26)	–
CZ	1.37 (1.11, 1.70)	0.619	9.8499 (29.47)	–
DE	1.02 (0.87, 1.23)	0.686	8.2046 (27.31)	–
DK	0.93 (0.75, 1.18)	0.669	5.2559 (11.62)	–
EE	1.07 (0.91, 1.28)	0.120	16.2827 (13.25)	–
ES	1.39 (1.21, 1.65)	0.501	15.1499 (23.92)	–
EU	1.34 (1.07, 1.73)	0.828	10.6031 (35.21)	–
FI	1.10 (0.80, 1.45)	0.930	11.0829 (20.20)	–
FR	0.66 (0.46, 0.96)	0.733	9.4318 (24.14)	–
GR	1.19 (1.06, 1.36)	0.757	12.4815 (20.68)	–
HR	0.79 (0.69, 0.97)	0.743	15.4879 (18.23)	-0.0791 (-1.89)
HU	0.96 (0.81, 1.20)	0.102	6.7687 (7.21)	–
IE	1.14 (1.00, 1.33)	0.435	4.9223 (8.02)	–
IT	0.69 (0.55, 0.88)	0.750	10.7022 (19.71)	–
LT	1.28 (1.09, 1.56)	0.492	16.8506 (18.84)	–
LU	0.38 (0.25, 0.54)	0.144	2.6636 (6.12)	0.0482 (5.39)
LV	1.29 (1.13, 1.50)	0.211	14.3487 (15.52)	–
MT	0.76 (0.61, 0.95)	0..035	6.5810 (10.74)	-0.0296 (-1.90)
NL	1.22 (1.00, 1.54)	0.714	3.3970 (10.74)	–
PL	1.27 (1.07, 1.56)	0.744	16.8843 (32.20)	–
PT	1.06 (0.93, 1.24)	0.511	4.5157 (7.99)	–
RO	0.65 (0.40, 1.04)	0.462	8.1390 (14.09)	-0.0382 (-2.06)
SE	1.05 (0.76, 1.46)	0.812	6.6881 (12.10)	–
SI	0.85 (0.71, 1.04)	0.543	7.0079 (13.71)	–
SK	1.31 (1.07, 1.70)	0.484	19.1342 (33.95)	–

The values in column 2 refer to the estimates of d (and 95% bands). In column 3, the estimate of the seasonal AR coefficient; in columns 3 and 4 the deterministic terms and their corresponding t-values.

Finally, we examine whether the Covid-19 pandemic has had any effect on the degree of persistence of the series of interest. For this purpose, we re-estimate the previous models using the seasonally unadjusted data but for the sample period ending in 2019Q4, that is, prior to the start of the pandemic. Table 7 reports the estimates of d for the three models considered, whilst Table 8 reports the estimated coefficients from the selected specifications, and Table 9 compares the estimates for the differencing parameter d and the seasonal AR coefficient obtained respectively for the period before the Covid-19 pandemic (i.e., ending in 2019Q4) and for the full sample including it (i.e., ending in 2020Q4).

**Table 7:** Seasonally unadjusted data. Results based on seasonal AR(1) errors.

Data ending at 2019Q4	No terms	A constant	A constant and a linear trend
AT	0.58 (0.42, 0.84)	**0.68 (0.51, 1.01)**	0.65 (0.51, 1.01)
BE	0.72 (0.50, 0.93)	**0.61 (0.46, 0.80)**	0.61 (0.46, 0.80)
BG	0.86 (0.62, 1.09)	0.94 (0.79, 1.18)	**0.95 (0.78, 1.19) (-)**
CY	0.99 (0.77 1.35)	**1.05 (0.88, 1.30)**	1.05 (0.88, 1.30)
CZ	0.85 (0.63, 1.09)	**1.37 (1.10, 1.71)**	1.37 (1.10, 1.70)
DE	0.89 (0.66, 1.13)	1.00 (0.87, 1.18)	**1.00 (0.86, 1.18) (-)**
DK	0.79 (0.60, 1.03)	**0.97 (0.79, 1.23)**	0.97 (0.79, 1.22)
EE	0.90 (0.71, 1.11)	**1.08 (0.91, 1.30)**	1.08 (0.92, 1.30)
ES	0.97 (0.77, 1.20)	**1.37 (1.20, 1.63)**	1.37 (1.20, 1.63)
EU	0.83 (0.59, 1.11)	**1.48 (1.26, 1.74)**	1.47 (1.26, 1.74)
FI	0.74 (0.49, 1.03)	**1.12 (0.85, 1.44)**	1.12 (0.86 1.43)
FR	0.73 (0.45, 1.02)	**1.11 (0.68, 1.46)**	1.11 (0.68, 1.44)
GR	0.97 (0.76, 1.26)	**1.32 (1.18, 1.53)**	1.32 (1.18, 1.55)
HR	0.93 (0.70, 1.25)	**0.76 (0.63, 0.94)**	0.75 (0.62, 0.94)
HU	0.88 (0.70, 1.16)	**0.97 (0.81, 1.23)**	0.97 (0.81, 1.23)
IE	1.09 (0.91, 1.30)	**1.23 (1.09, 1.43)**	1.23 (1.09, 1.43)
IT	0.83 (0.58, 1.15)	**0.99 (0.79, 1.26)**	0.99 (0.79, 1.25)
LT	0.98 (0.78, 1.22)	**1.28 (1.09, 1.56)**	1.28 (1.09, 1.56)
LU	0.28 (0.21, 0.48)	**0.48 (0.39, 0.59)**	0.37 (0.23, 0.54)
LV	1.03 (0.85, 1.26)	**1.29 (1.12, 1.51)**	1.29 (1.12, 1.51)
MT	0.92 (0.66, 1.13)	0.74 (0.62, 0.92)	**0.71 (0.57, 0.91) (-)**
NL	0.91 (0.69, 1.19)	**1.26 (1.04, 1.56)**	1.26 (1.04, 1.56)
PL	0.87 (0.67, 1.14)	**1.28 (1.09, 1.57)**	1.28 (1.09, 1.57)
PT	1.02 (0.82, 1.27)	**1.27 (1.10, 1.51)**	1.27 (1.10, 1.51)
RO	0.71 (0.44, 1.00)	0.56 (0.39, 1.01)	**0.58 (0.27, 1.02) (-)**
SE	0.67 (0.42, 0.96)	1.05 (0.78, 1.38)	**1.05 (0.77, 1.37) (-)**
SI	0.78 (0.54, 1.05)	**0.85 (0.69, 1.08)**	0.85 (0.69, 1.08)
SK	0.93 (0.68, 1.18)	**1.29 (1.04, 1.68)**	1.29 (1.04, 1.68)

The reported values are the estimates of d. In parenthesis, the 95% confidence intervals. In bold, the selected specification according to the deterministic terms. ∗: evidence of mean reversion at the 95% level; (+) indicates a positive time trend and (-) a negative time trend.

**Table 8:** Estimated coefficients from the selected models in Table 7.

Country	d	Seasonal	Intercept	Time trend
AT	0.68 (0.51, 1.01)	0.428	4.3675 (11.48)	–
BE	0.61 (0.46, 0.80)	0.444	7.2754 (19.29)	–
BG	0.95 (0.78, 1.19)	0.472	18.8091 (18.17)	-0.1801 (-1.78)
CY	1.05 (0.88, 1.30)	0.685	5.5328 (7.17)	–
CZ	1.37 (1.10, 1.71)	0.616	9.8467 (28.66)	–
DE	1.00 (0.86, 1.18)	0.696	8.2661 (27.38)	-0.0662 (-1.86)
DK	0.97 (0.79, 1.23)	0.693	5.2779 (11.95)	–
EE	1.08 (0.91, 1.30)	0.176	16.3072 (13.09)	–
ES	1.37 (1.20, 1.63)	0.479	15.1415 (23.42)	–
EU	1.48 (1.26, 1.74)	0.878	10.6485 (46.38)	–
FI	1.12 (0.85, 1.44)	0.942	11.0944 (23.09)	–
FR	1.11 (0.68, 1.46)	0.838	10.3395 (30.11)	–
GR	1.32 (1.18, 1.53)	0.801	12.5695 (24.29)	–
HR	0.76 (0.63, 0.94)	0.742	15.1667 (16.97)	–
HU	0.97 (0.81, 1.23)	0.105	6.7769 (6.74)	–
IE	1.23 (1.09, 1.43)	0.421	4.9336 (9.02)	–
IT	0.99 (0.79, 1.26)	0.830	11.3886 (25.38)	–
LT	1.28 (1.09, 1.56)	0.488	16.8522 (18.27)	–
LU	0.48 (0.39, 0.59)	0.254	4.0640 (12.71)	–
LV	1.29 (1.12, 1.51)	0.210	14.3493 (14.83)	–
MT	0.71 (0.57, 0.91)	0.035	6.6503 (18.81)	-0.0320 (-2.11)
NL	1.26 (1.04, 1.56)	0.749	3.4130 (11.29)	–
PL	1.28 (1.09, 1.57)	0.752	16.8873 (31.18)	–
PT	1.27 (1.10, 1.51)	0.571	4.6010 (10.09)	–
RO	0.58 (0.27, 1.02)	0.456	8.0461 (14.50)	-0.0350 (-2.04)
SE	1.05 (0.77, 1.37)	0.827	6.8689 (13.78)	–
SI	0.85 (0.69, 1.08)	0.474	7.0164 (13.36)	–
SK	1.29 (1.04, 1.68)	0.534	19.1357 (31.85)	–

The values in column 2 refer to the estimates of d (and 95% bands). In column 3, the estimate of the seasonal AR coefficient; in columns 3 and 4 the deterministic terms and their corresponding t-values.

**Table 9:** Comparison -2019 with -2020.

Country	d		Seasonality	
	-2019	-2020	-2019	-2020
AT	0.68 (0.51, 1.01)	0.70 (0.50, 1.02)	0.428	0.418
BE	0.61 (0.46, 0.80)	0.66 (0.54, 0.81)	0.444	0.452
BG	0.95 (0.78, 1.19)	0.90 (0.75, 1.12)	0.472	0.471
CY	1.05 (0.88, 1.30)	1.06 (0.89, 1.32)	0.685	0.729
CZ	1.37 (1.10, 1.71)	1.37 (1.11, 1.70)	0.616	0.619
DE	1.00 (0.86, 1.18)	1.02 (0.87, 1.23)	0.696	0.686
DK	0.97 (0.79, 1.23)	0.93 (0.75, 1.18)	0.693	0.669
EE	1.08 (0.91, 1.30)	1.07 (0.91, 1.28)	0.176	0.120
ES	1.37 (1.20, 1.63)	1.39 (1.21, 1.65)	0.479	0.501
EU	1.48 (1.26, 1.74)	1.34 (1.07, 1.73)	0.878	0.828
FI	1.12 (0.85, 1.44)	1.10 (0.80, 1.45)	0.942	0.930
FR	1.11 (0.68, 1.46)	0.66 (0.46, 0.96)	0.838	0.733
GR	1.32 (1.18, 1.53)	1.19 (1.06, 1.36)	0.801	0.757
HR	0.76 (0.63, 0.94)	0.79 (0.69, 0.97)	0.742	0.743
HU	0.97 (0.81, 1.23)	0.96 (0.81, 1.20)	0.105	0.102
IE	1.23 (1.09, 1.43)	1.14 (1.00, 1.33)	0.421	0.435
IT	0.99 (0.79, 1.26)	0.69 (0.55, 0.88)	0.830	0.750
LT	1.28 (1.09, 1.56)	1.28 (1.09, 1.56)	0.488	0.492
LU	0.48 (0.39, 0.59)	0.38 (0.25, 0.54)	0.254	0.144
LV	1.29 (1.12, 1.51)	1.29 (1.13, 1.50)	0.210	0.211
MT	0.71 (0.57, 0.91)	0.76 (0.61, 0.95)	0.035	0.035
NL	1.26 (1.04, 1.56)	1.22 (1.00, 1.54)	0.749	0.714
PL	1.28 (1.09, 1.57)	1.27 (1.07, 1.56)	0.752	0.744
PT	1.27 (1.10, 1.51)	1.06 (0.93, 1.24)	0.571	0.511
RO	0.58 (0.27, 1.02)	0.65 (0.40, 1.04)	0.456	0.462
SE	1.05 (0.77, 1.37)	1.05 (0.76, 1.46)	0.827	0.812
SI	0.85 (0.69, 1.08)	0.85 (0.71, 1.04)	0.474	0.543
SK	1.29 (1.04, 1.68)	1.31 (1.07, 1.70)	0.534	0.484

The values in columns 2 and 3 refer to the estimates of d (and 95% bands). In columns 4 and 5, the estimates of the seasonal AR coefficient.

It can be seen that the full-sample estimates of d are higher than those for the shorter sample excluding the pandemic period in the case of 10 countries, namely Austria, Belgium, the Czech Republic, Germany, Spain, Croatia, Lithuania, Malta, Romania and Slovakia, whilst they are lower in the case of the EU mean series and of 13 countries, specifically Bulgaria, Denmark, Estonia, Finland, France, Greece, Hungary, Ireland, Italy, Luxembourg, Netherlands, Poland and Portugal, and they are the same in the remaining four countries, i.e. Cyprus, Latvia, Sweden and Slovenia. As for the degree of seasonal persistence, this is found to be higher when considering the full sample in the case of 10 countries ten while it decreases in 16 countries as well as the EU mean series, with only Malta displaying the same seasonal AR coefficient whichever sample is used for the estimation.

## 5 Conclusions

This paper investigates unemployment persistence in the 27 EU member states applying fractional integration methods to quarterly data (both seasonally adjusted and unadjusted) from 2000q1 to 2020q4. On the whole, the evidence points to high levels of persistence in the unemployment rates of all the 27 countries examined. With seasonally adjusted data, a small degree of mean reversion is found in the case of Belgium, Luxembourg and Malta, but this evidence disappears under the assumption of weakly correlated disturbances. More cases of mean reversion are found instead when analyzing the unadjusted series. In particular, countries such as Belgium, France, Croatia, Italy, Luxembourg and Malta display orders of integration significantly lower than 1. In addition, significant negative time trends are found in the case of Bulgaria, Croatia, Malta and Romania, and a positive one for Luxembourg. Finally, the Covid-19 pandemic had mixed effects, with (seasonal) persistence increasing in some countries whilst decreasing in others and having no impact in a minority of cases.

On the whole, our results support the hysteresis hypothesis for the European economies, rather than models in which there is convergence towards a long-run equilibrium level, be it the neoclassical natural rate or the New Keynesian NAIRU. consistently with previous studies such as those by Blanchard and Summers (1986), Alogoskoufis and Manning (1988), Caporale and Gil-Alana (2008), Cuestas et al. (2011). However, in some countries such as Belgium, Luxembourg and Malta the speed of adjustment towards the long-run equilibrium is relatively fast. There are several reasons that might explain why unemployment persistence is particularly high in Europe. One of them is the role of unions, which are bigger in Europe than anywhere else, and reduce the range of policy measures that can be adopted in response to shocks. The existence of unemployment benefits is another factor to take into consideration. Benefits (and the higher minimum wage in Europe than elsewhere) increase the reservation wage of workers, thus making them more reluctant to accept the lower wage jobs available during depressions. Psychological factors such as the stigma of being a long-term unemployed can also make companies less likely to hire these workers. Finally, the high average age of employees (resulting from the high life expectancy in Europe) also plays since it is more difficult for older workers to adapt to new technologies.

## Declarations

### Author contribution statement

Guglielmo Maria Caporale, Luis A. Gil-Alana and Pablo Vicente Trejo: Conceived and designed the experiments; Performed the experiments; Analyzed and interpreted the data; Contributed reagents, materials, analysis tools or data; Wrote the paper.

### Funding statement

This work was supported by the MINEIC-AEI-FEDER PID2020-113691RB-I00 project from "10.13039/501100010198Ministerio de Economía, Industria y Competitividad" (MINEIC-AEI-FEDER PID2020-113691RB-I00), "Agencia Estatal de Investigación" (AEI) Spain and "Fondo Europeo de Desarrollo Regional" (FEDER), and from an internal project of the 10.13039/100012986Universidad Francisco de Vitoria.

### Data availability statement

Data will be made available on request.

### Declaration of interests statement

The authors declare no conflict of interest.

### Additional information

No additional information is available for this paper.
